# RETRACTION: Effect of Instant Surgery Compared with Traditional Management on Paediatric Complicated Acute Appendicitis Post‐Surgery Wound: A Meta‐Analysis

**DOI:** 10.1111/iwj.70220

**Published:** 2025-02-11

## Abstract

**RETRACTION**: CaoX. , GengX. , ZhangC. , ChenJ. , ZhangC. , LiuQ. , WuT. , and LiL. , “Effect of Instant Surgery Compared with Traditional Management on Paediatric Complicated Acute Appendicitis Post‐Surgery Wound: A Meta‐Analysis,” International Wound Journal 20, no. 8 (2023): 2964–2972, 10.1111/iwj.14163.36965159 PMC10502279

The above article, published online on 25 March 2023, in Wiley Online Library (http://onlinelibrary.wiley.com/), has been retracted by agreement between the journal Editor in Chief, Professor Keith Harding; and John Wiley & Sons Ltd. Following an investigation by the publisher, all parties have concluded that this article was accepted solely on the basis of a compromised peer review process. The editors have therefore decided to retract the article. The authors did not respond to our notice regarding the retraction.

**RETRACTION**: X. Cao , X. Geng , C. Zhang , J. Chen , C. Zhang , Q. Liu , T. Wu , and L. Li , “Effect of Instant Surgery Compared with Traditional Management on Paediatric Complicated Acute Appendicitis Post‐Surgery Wound: A Meta‐Analysis,” International Wound Journal 20, no. 8 (2023): 2964–2972, 10.1111/iwj.14163.36965159 PMC10502279

The above article, published online on 25 March 2023, in Wiley Online Library (http://onlinelibrary.wiley.com/), has been retracted by agreement between the journal Editor in Chief, Professor Keith Harding; and John Wiley & Sons Ltd. Following an investigation by the publisher, all parties have concluded that this article was accepted solely on the basis of a compromised peer review process. The editors have therefore decided to retract the article. The authors did not respond to our notice regarding the retraction.

